# Weightbearing Computed Tomography (WBCT) Analysis of Subtalar Joint Dynamics in Hindfoot Valgus Malalignment

**DOI:** 10.3390/jcm14082587

**Published:** 2025-04-09

**Authors:** Otgonsaikhan Nomkhondorj, Dong-Il Chun, Kwang-Rak Park, Jaeho Cho

**Affiliations:** 1 Department of and Biomedical Science, Graduate School of Medicine, Hallym University, Chuncheon 24252, Republic of Korea; otgonsaikhan0899@gmail.com; 2 Department of Orthopaedic Surgery, Chuncheon Sacred Heart Hospital, Hallym University, Chuncheon 24252, Republic of Korea; 3 Department of Orthopaedic Surgery, Soonchunhyang University Seoul Hospital, Seoul 04401, Republic of Korea; orthochun@gmail.com; 4 Department of Anatomy, College of Korean Medicine, Sangji University, Wonju 26339, Republic of Korea; airboba@sangji.ac.kr

**Keywords:** hindfoot, alignment, weightbearing, computed tomography, impingement, advanced, image

## Abstract

**Background/Objectives:** Hindfoot valgus malalignment, characterized by the lateral deviation of the calcaneus and medial tilting of the talus, disrupts hindfoot biomechanics and increases strain on subtalar joint. This study evaluates weightbearing and non-weightbearing imaging modalities to identify dynamic alignment changes and their diagnostic implications. This study aims to (1) quantify changes in subtalar joint parameters between non-weightbearing computed tomography (NWBCT) and weightbearing computed tomography (WBCT) in patients with hindfoot valgus; (2) evaluate correlations between WBCT and standard radiographic parameters; and (3) identify radiographic predictors of subtalar joint status during weightbearing. **Methods:** We reviewed 70 patients with confirmed hindfoot valgus malalignment (hindfoot valgus angle >5°), identified through radiographic measurements. Of these, 32 underwent both NWBCT and WBCT, while 38 underwent WBCT alone. Hindfoot alignment angle (HAA) and hindfoot alignment ratio (HAR) were measured on hindfoot alignment radiographs, while heel valgus angle (HVA), talocalcaneal distance (TCD), subtalar joint subluxation (SL) and calcaneofibular distance (CF) were assessed on CT. **Results:** WBCT revealed significant increases in HVA and SL (both, *p* < 0.001) and decreases in TCD and CF (*p* < 0.001 and *p* = 0.002, respectively) compared to NWBCT, reflecting dynamic subtalar joint changes under weightbearing conditions. Receiver operating characteristic (ROC) analysis identified hindfoot alignment angle (HAA) as the most reliable predictor of talocalcaneal osseous contact, with a cutoff value of >9.25° based on Youden’s index, yielding a sensitivity of 73% and specificity of 81.8%. Inter- and intra-observer reliabilities for all parameters were excellent (ICC > 0.81). **Conclusions:** WBCT provides critical insights into subtalar joint dynamics under physiological loads, surpassing NWBCT in assessing weightbearing-induced alignment changes. Although standard radiographic parameters, particularly HAA, can serve as reliable, cost-effective predictors of subtalar joint pathology in resource-limited settings, WBCT should still be preferred when available, especially in patients with significant malalignment or when detailed dynamic evaluation is needed to guide clinical decision-making.

## 1. Introduction

Hindfoot valgus malalignment, characterized by lateral deviation of the calcaneus and medial tilting of the talus, disrupts normal hindfoot mechanics and places excessive strain on medial soft tissues, contributing to instability, joint degeneration and chronic pain. This condition, which affects approximately 4–8% of the general population, is frequently associated with adult-acquired flatfoot deformity (AAFD), posterior tibial tendon dysfunction (PTTD) and other pathologies affecting the stability and function of the subtalar joint [1,2,3]. Clinically, hindfoot valgus is defined as a deformity with a hindfoot valgus angle >5°, measured on radiographs or advanced imaging modalities [1,2,3]. Over time, this bio-mechanical dysfunction can cause sinus tarsi narrowing, talocalcaneal impingement and progressive joint degeneration, resulting in pain, instability and restricted mobility [4,5]. Hindfoot valgus can be both a cause and a consequence of conditions like AAFD and PTTD, depending on the stage of the disease. It is important to differentiate hindfoot valgus malalignment from subtalar joint assessment, as these two represent distinct aspects of foot pathology. Hindfoot valgus malalignment primarily refers to the misalignment of the hindfoot relative to the tibial axis, while subtalar joint assessment focuses on the functional state of the joint, which can be influenced by factors such as subluxation or impingement.

The accurate assessment of hindfoot valgus malalignment and its associated pathologies is critical for effective diagnosis and treatment planning, with imaging modalities playing a central role in evaluation. Standard radiographs, including hindfoot alignment views, are widely used for initial assessment due to their accessibility and cost-effectiveness. However, their limitations include the superimposition of bony structures and inability to evaluate functional changes under load [6,7]. Conventional non-weightbearing computed tomography (NWBCT) offers the superior visualization of bony structures and joint congruence, making it a valuable tool for detailed anatomical assessment. Despite these advantages, NWBCT does not replicate the functional loading conditions of the foot, which are essential for understanding dynamic changes in alignment and joint interactions under physiologic load [8,9,10]. As a result, NWBCT may underestimate the extent of deformity and its impact on the hindfoot.

Weightbearing computed tomography (WBCT) has emerged as a powerful tool for assessing hindfoot deformities under physiological loads. By capturing three-dimensional images of the foot in a standing position, WBCT provides a more realistic representation of joint relationships and alignment changes. This technique is particularly valuable for evaluating the subtalar joint, where subtle shifts in alignment under load may reveal critical information about joint instability, subluxation and impingement that would otherwise remain undetected in non-weightbearing imaging [11,12,13,14,15]. Recent studies have demonstrated the utility of WBCT in assessing subtalar joint dynamics and its potential impact on clinical decision-making, especially when evaluating complex deformities such as hindfoot valgus malalignment [16,17]. Understanding how weightbearing influences joint dynamics is crucial for quantifying the severity of deformity and its impact on adjacent structures. To date, the only study investigating the relationship between hindfoot alignment observed on plain radiographs and subtalar joint alignment observed on WBCT has been conducted in patients with progressive collapsing foot deformity (PCFD). This gap in knowledge regarding other types of hindfoot valgus malalignment warrants further investigation [18,19]. Furthermore, WBCT is not universally available in orthopedic centers, necessitating accessible alternatives. Identifying radiographic parameters from standard radiographs that correlate with subtalar joint changes could offer cost-effective diagnostic tools, reducing reliance on advanced imaging technologies in resource-limited settings.

The purpose of this study was to (1) quantify the changes in subtalar joint parameters between NWBCT and WBCT in patients with hindfoot valgus; (2) assess the correlation between radiographic parameters of the subtalar joint in WBCT and hindfoot alignment measured in standard radiographic views; and (3) determine whether specific radiographic parameters can predict the status of the subtalar joint during weightbearing using hindfoot valgus alignment observed in simple radiographs.

## 2. Materials and Methods

This study was a retrospective, cross-sectional observational study conducted between 1 January 2024 and 30 November 2024. This study protocol was reviewed and approved by the institutional review board (CHUNCHEON 2024-12-003), and all patients provided informed consent.

### 2.1. Patient Selection

Between 1 January 2024 and 30 November 2024, patients who underwent WBCT scans (Planmed Verity Extremity, Planmed Oy, Helsinki, Finland) at our institution were reviewed for inclusion in this study. Hindfoot valgus malalignment was defined as a hindfoot valgus angle greater than 5° on hindfoot alignment view. The hindfoot alignment view is a radiographic projection (20 degrees) obtained to measure the hindfoot angle, typically an anteroposterior (AP) view of the foot that allows the assessment of alignment between the tibia and calcaneus. Hindfoot valgus malalignment was assessed in patients with suspected or diagnosed foot deformities, including adult-acquired flatfoot deformity, and those presenting with foot pain, instability, or functional impairment. Patients were symptomatic and exhibited clinical signs such as pain or instability in the foot and ankle. Inclusion criteria required confirmed hindfoot valgus alignment on radiographic measurement. Exclusion criteria included patients under 18 years of age, a history of prior foot or ankle surgery, advanced arthritis of the foot or ankle and structural deformities such as bone dysplasia.

A total of 70 patients (35 women and 35 men; mean age of 39.6 years; range of 18–69 years) met the inclusion criteria for this study. Among them, 32 patients (14 women and 18 men; mean age of 41.3 years; range of 18–65 years) underwent both non-weightbearing computed tomography (NWBCT) and WBCT scans. These patients were included to quantify changes in subtalar joint parameters between NWBCT and WBCT. An additional 38 patients with hindfoot valgus malalignment who underwent WBCT but not NWBCT were included for analysis, resulting in a combined cohort of 70 patients. The subgroup of 32 patients underwent both NWBCT and WBCT to compare changes in subtalar joint parameters between the two imaging techniques, while the remaining 38 patients underwent only WBCT, as they were part of a larger cohort to assess WBCT’s diagnostic capability in isolating subtalar joint malalignment in patients with hindfoot valgus.

### 2.2. Imaging Techniques

**Figure 1:**
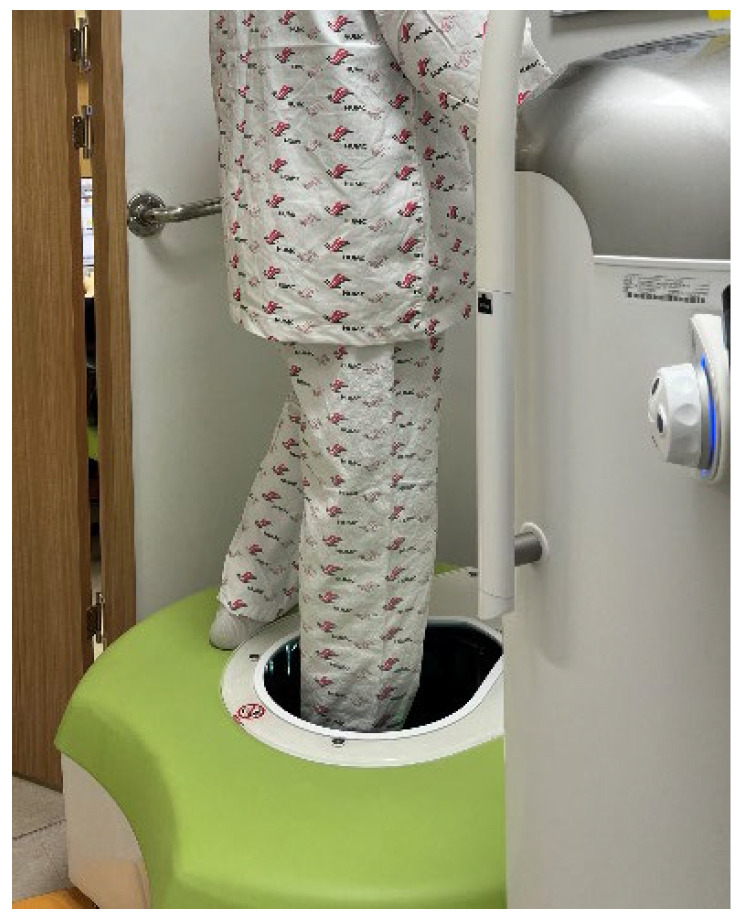
This photograph showed the position during upright weightbearing foot examination using a cone-beam CT scanner. Scanner design with flexible gantry movements allows weightbearing imaging in a standing position.

For NWBCT and WBCT, specific positioning protocols were followed to standardize patient posture and minimize motion artifacts, ensuring reliable imaging for subsequent radiographic analysis. For NWBCT, patients were seated with the ankle in a neutral position, stabilized using a short leg splint at 90 degrees to prevent motion artifacts [16]. For WBCT, patients stood with their foot in a neutral position on a dedicated platform, with the other foot resting on the gantry. The horizontal orientation of the gantry allowed imaging close to floor level, enabling weightbearing scans (Figure 1).

### 2.3. Radiographic Measurements

**Figure 2:**
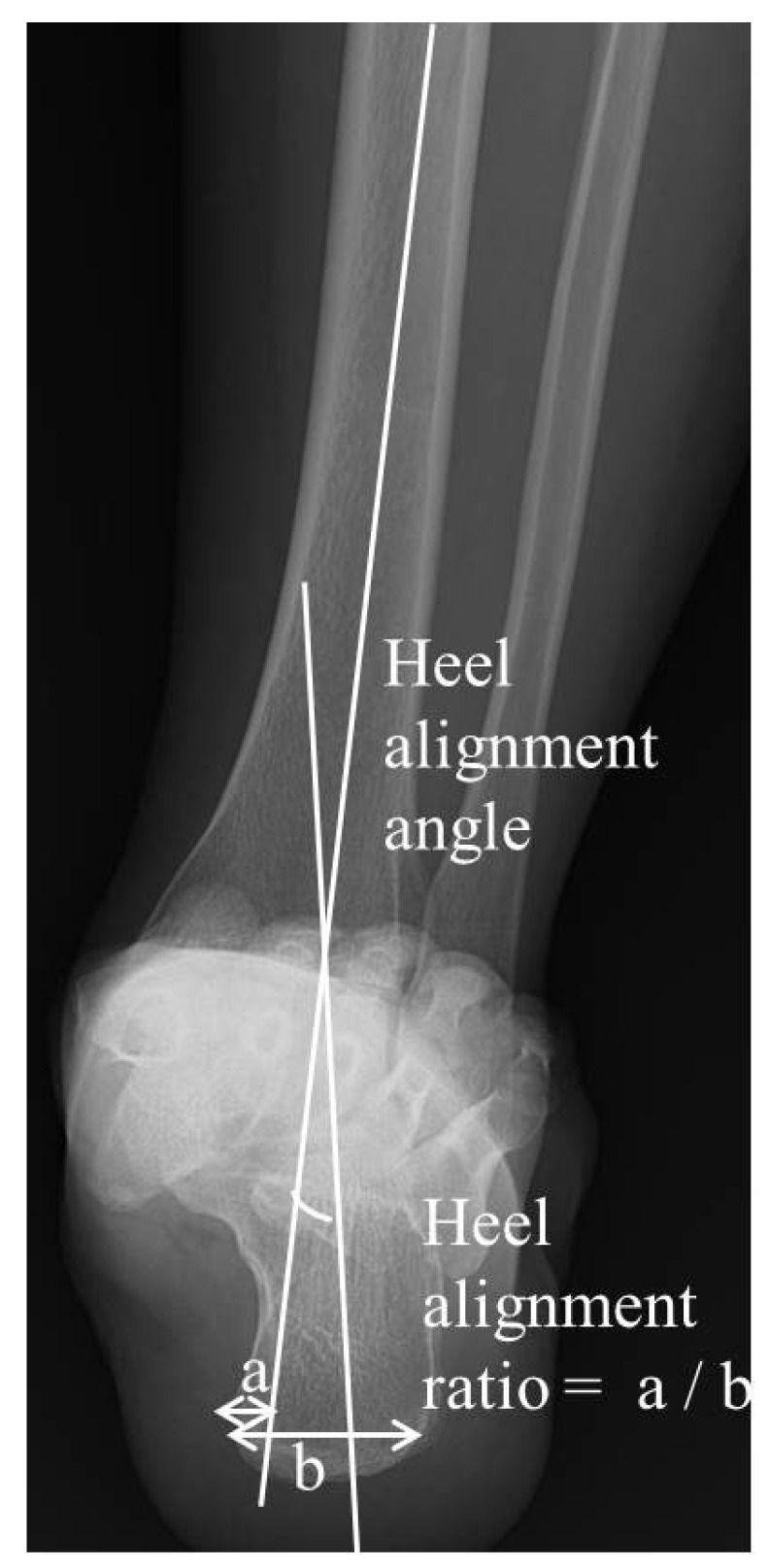
In the hindfoot alignment view, hindfoot alignment angle and hindfoot alignment ratio were measured.

Radiographic measurements were derived from the obtained hindfoot alignment view, NWBCT and WBCT scans, focusing on hindfoot and subtalar joint parameters using defined anatomical landmarks and angles. The hindfoot alignment view is a radiographic projection (20 degrees) obtained to measure the hindfoot angle during weightbearing, typically an anteroposterior (AP) view of the foot that allows the assessment of alignment between the tibia and calcaneus. To assess the hindfoot alignment in a simple radiograph, two measurements were obtained for the hindfoot alignment view: (1) the hindfoot alignment angle (HAA) and (2) the hindfoot alignment ratio (HAR) [20,21]. The hindfoot alignment angle (HAA), which is an angle between the tibial axis and calcaneal axis, was measured and expressed as a positive number when it was in valgus. The hindfoot alignment ratio (HAR) was obtained by dividing the width of the calcaneus medial to the tibial axis by calcaneal width at its widest portion on the alignment view (Figure 2). The tibial axis was defined using a line perpendicular to the distal tibial joint surface, while the calcaneal axis was determined by drawing a line along the medial contour of the calcaneus on the most posterior image that includes both the tibia and calcaneus.

### 2.4. Subtalar Joint Parameters on CT

**Figure 3:**
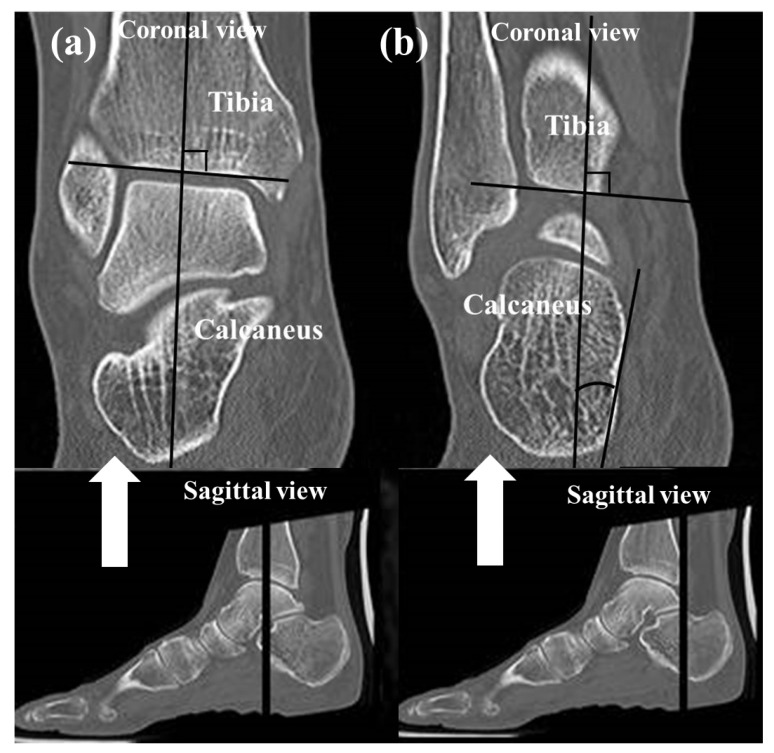
Coronal images of the ankle joint show the measurement technique for the heel valgus angle. (**a**) The axis of the distal tibia is defined by a perpendicular line to the midportion of the distal tibial joint surface. (**b**) Measurement was obtained on the most posterior image including the tibia and calcaneus between the tibial axis and a line adapted to the medial osseous contour of the calcaneus.

**Figure 4:**
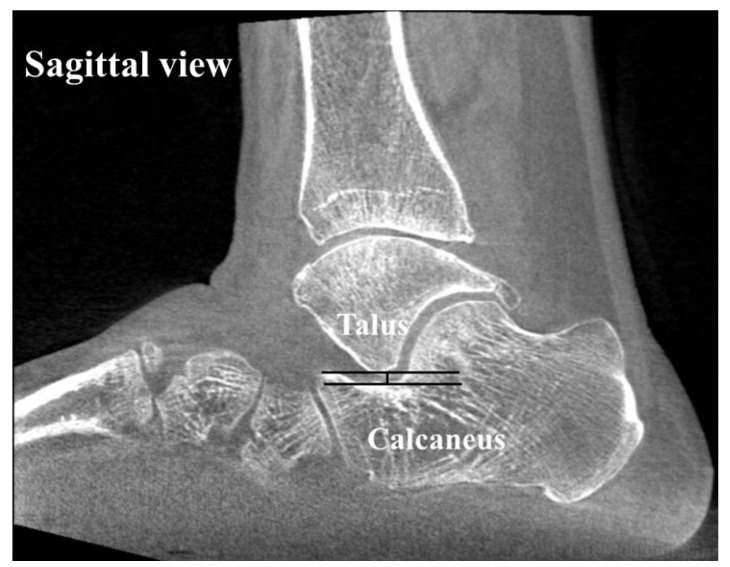
The sagittal image of the foot shows the measurement for the talocalcaneal distance at the Gissane angle. Measurement was assessed as the shortest distance from the most inferior aspect of lateral talus process to the floor of the calcaneus in the sinus tarsi.

**Figure 5:**
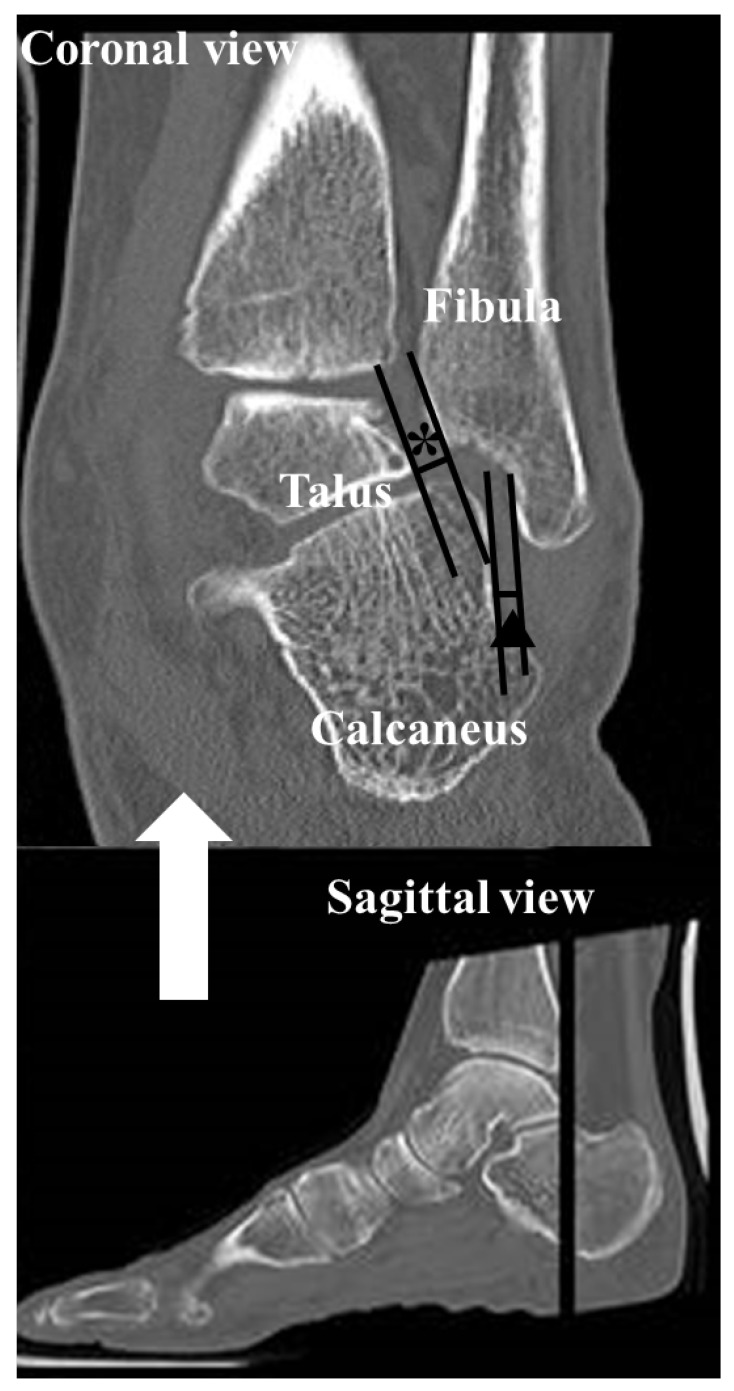
The subtalar joint subluxation (*) and the calcaneofibular distance (▲) were measured in the coronal image at the level of the most posterior aspect of the fibula. The subtalar joint subluxation was measured from the lateral margin of the calcaneal articular surface to the lateral margin of the talar articular surface. The calcaneofibular distance was measured from the lateral margin of the calcaneal wall surface to the medial margin of the fibular articular surface.

To assess the alignment of the hindfoot, including the subtalar joint on CT, four measurements which have been used in previous studies [1,22,23,24,25] were used: (1) the heel valgus angle (HVA), (2) the talocalcaneal distance (TCD) at the Gissane angle, (3) the subtalar joint subluxation (SL) and (4) the calcaneofibular distance (CF). Axial, sagittal and coronal (reconstruction thickness of 2 mm) bone window reformations of axial images were used for measurements. The heel valgus angle (HVA) was based on the axis of the distal tibial defined by a perpendicular line to the distal tibia joint surface and a line parallel the medial osseous contour of the calcaneus on the most posterior image, including the tibia and calcaneus (Figure 3). Axis of the distal tibia was defined using the most central coronal image through the distal tibial shaft. This image was identified as the one in which the tibial shaft diameter was maximal, and the tibial cortex was sharply defined. The talocalcaneal distance (TCD) at the Gissane angle was assessed measuring the shortest distance from the most inferior aspect of lateral talus process to the floor of the calcaneus in the sinus tarsi. This measurement was seen on sagittal images (Figure 4). The subtalar joint subluxation (SL) and the calcaneofibular distance (CF) were measured in the coronal plane at the level of the most posterior aspect of the fibula (Figure 5). The subtalar joint subluxation (SL) was measured from the lateral margin of the calcaneal articular surface to the lateral margin of the talar articular surface. The calcaneofibular distance (CF) was measured from the lateral margin of the calcaneal wall surface to the medial margin of the fibular articular surface.

For the subgroup of 32 patients, these parameters of the hindfoot with the subtalar joint on CT were compared between NWBCT and WBCT to determine the effect of weightbearing on the subtalar joint alignment. For the larger cohort of 70 patients, correlations between subtalar joint parameters measured on WBCT and hindfoot alignment derived from standard radiographic images were analyzed to predict talocalcaneal contact in patients with hindfoot valgus malalignment. The receiver-operating statistics analysis (ROC) curve was analyzed to determine the optimal cutoff value for hindfoot alignment angle (HAA) to predict sinus tarsi bony impingement. Sensitivity, specificity and the area under the curve (AUC) were calculated to evaluate diagnostic performance.

### 2.5. Statistical Analysis

All radiographs were reviewed independently by two authors (O.N. and J.C.). Both observers (O.N. and J.C.) are experienced foot and ankle surgeons specializing in orthopedic radiology. Inter- and intra-observer reliabilities were obtained for all radiographic parameters using the intraclass correlation coefficient (ICC). According to the definitions of Landis and Koch [26], an ICC of 0.81–1.00 was interpreted as excellent, 0.61–0.80 as good, 0.41–0.60 as moderate, 0.21–0.40 as fair and 0.00–0.20 as poor. The intra-class correlation coefficient (ICC) was determined using two separate assessments of radiographic measurements performed by both observers. Intra-observer variability was calculated by having each observer repeat measurements on a separate occasion, with a time interval of two weeks between the initial and subsequent measurements. The *p*-value of <0.05 was considered statistically significant. Descriptive statistics were used to report quantitative data. The Wilcoxon signed rank test was used to assess the changes between NWBCT and WBCT. The Pearson correlation coefficient (PCC) was calculated to quantify the correlation between radiographic parameters of subtalar joint in the WBCT and alignment in the hindfoot alignment view. The ROC analysis was conducted to assess the predictive value of hindfoot alignment angle (HAA) and hindfoot alignment ratio (HAR) for talocalcaneal contact (dependent variable) at the Gissane angle, which was indicative of sinus tarsi bony impingement. A threshold value was determined based on optimized sensitivity and specificity to distinguish between patients with and without talocalcaneal contact. Data were analyzed using IBM SPSS Statistics, version 23.0, for Windows (IBM Co., Armonk, NY, USA).

## 3. Results

The reliability statistics for inter- and intra-observer comparisons are shown in Table 1. Inter- and intra-observer reliability were very high regarding all radiographic parameters. In all 70 patients with hindfoot valgus malalignment, the mean HAA was 9.6 ± 4.0° (range, 6 to 21°) and the mean HAR was 0.12 ± 0.25 (range, −0.44 to 0.49).

The alignment of the hindfoot, including the subtalar joint, changed significantly in the upright weightbearing CT position in 32 patients with hindfoot valgus malalignment. Significant differences were observed in all measurements between NWBCT and WBCT (Table 2). The mean HVA and mean SL increased significantly in WBCT compared to NWBCT (both, *p* < 0.001). Also, the mean TCD at the Gissane angle and the mean CF decreased significantly in WBCT compared to NWBCT (*p* < 0.001 and *p* = 0.002, respectively).

The results of Pearson’s statistics to assess the correlation between radiographic parameters of subtalar joint in the WBCT and alignment in the hindfoot alignment view are provided in Table 3. The HVA and TCD at the Gissane angle in WBCT had statistically significant positive correlation of two measurements (HAA and HAR) in the hindfoot alignment view.

**Figure 6:**
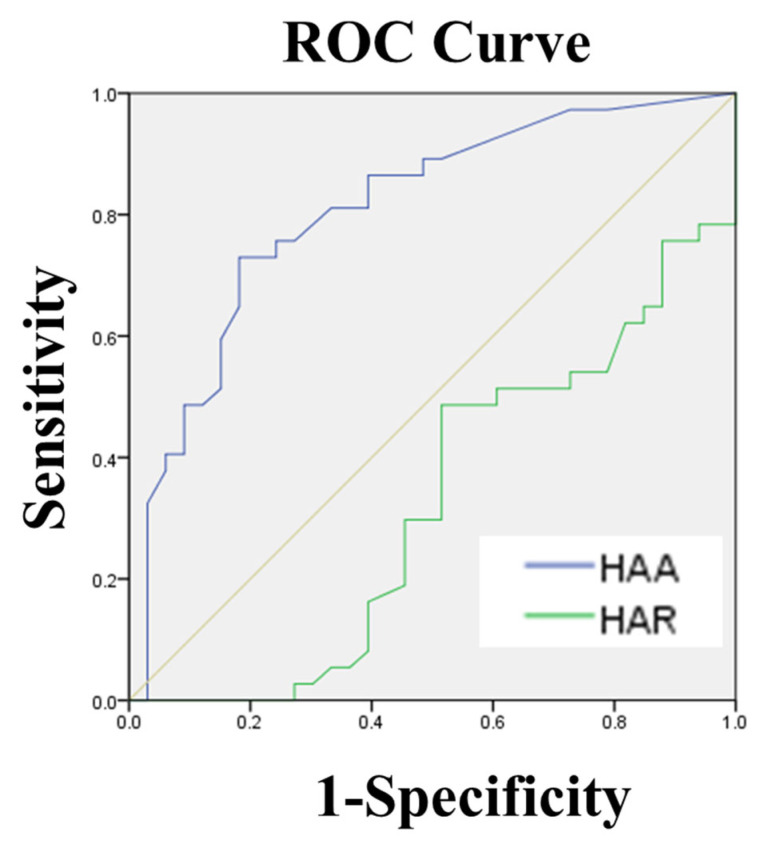
Receiver operating characteristic (ROC) curve showing the efficiency of the parameters in standard radiographs for the prediction of contact condition between the talus and calcaneus at the Gissane angle. The area under curve (AUC) values of the hindfoot alignment angle (HAA) and the hindfoot alignment ratio (HAR) were 0.807 and 0.323, respectively.

Thus, we determined the talocalcaneal distance (TCD) at the Gissane angle as the radiographic parameter of subtalar joint during upright weightbearing, which could be be predicted by measuring the hindfoot valgus alignment in simple radiograph. Referencing prior cadaver study [27], we postulated that the talocalcaneal osseous contact was less than 2 mm of talocalcaneal distance at the Gissane angle in WBCT. In the ROC curve analysis, hindfoot alignment angle (HAA) had the greater AUC (area under the curve = 0.807, 95% CI = 0.703–0.911) compared with the hindfoot alignment ratio (HAR) (area under the curve = 0.323, 95% CI = 0.197–0.449), suggesting that HAA is the best radiographic parameter for predicting the contact condition between talus and calcaneus at the Gissane angle (sinus tarsi bony impingement) [28]. The ROC curve analysis identified a threshold HAA value of 9.25°, achieving 73.0% sensitivity and 81.8% specificity for predicting talocalcaneal contact, with an AUC of 0.807 (Figure 6).

## 4. Discussion

Conventional weightbearing ankle and hindfoot radiographs are useful for assessing hindfoot valgus alignment. However, the superimposition of tarsal bones in X-rays limits the precise evaluation of the subtalar joint [29]. Cross-sectional imaging, such as CT and MRI, is superior to radiographs in assessing subtalar joint abnormalities and has been widely utilized to evaluate foot deformities and hindfoot impingement associated with the hindfoot valgus. However, conventional CT and MRI are limited by their supine imaging positions [25,30,31]. The novel cone-beam extremity CT allows upright weightbearing scans of the lower extremity which provides three-dimensional images of foot structures and intertarsal relationships under physiological, weightbearing conditions [16]. Furthermore, cone-beam CT offers the advantage of significantly lower radiation exposure compared to conventional CT. The effective radiation dose for lower extremity cone-beam CT has been reported to range from approximately 2 to 6 µSv, whereas a standard chest X-ray typically delivers about 50 to 100 µSv, and conventional multi-slice CT scans of the lower extremity may exceed 1000 µSv, depending on the imaging protocol [32]. This makes it a safer imaging modality for assessing hindfoot alignment, especially when both NWBCT and WBCT scans are required for comparative analysis. In our study, both imaging acquisitions were performed using the same cone-beam CT system, minimizing additional radiation exposure while maintaining diagnostic accuracy [33].

The findings of this study highlight significant differences in all measured parameters related to hindfoot alignment, including subtalar joint alignment, between non-weightbearing CT (NWBCT) and weightbearing CT (WBCT) in patients with hindfoot valgus malalignment. These differences underscore the critical role of weightbearing in influencing subtalar joint alignment. Specifically, the heel valgus angle (HVA) and subtalar joint subluxation (SL) were notably higher in WBCT images compared to NWBCT, reflecting an increased talar tilt and calcaneal lateral deviation under load. Conversely, the talocalcaneal distance (TCD) at the Gissane angle and the calcaneofibular distance (CF) were reduced in the weightbearing condition, indicating joint space compaction and closer osseous structure approximation due to physiological load. The pronounced hindfoot valgus observed in the weightbearing position may account for these significant changes in subtalar joint alignment compared to the non-weightbearing position. This difference in alignment between WBCT and NWBCT is especially relevant when comparing results in healthy populations, where weightbearing forces can cause a more significant shift in joint position. While NWBCT provides a static view of the anatomy without considering the effects of weightbearing, it is limited in capturing the full range of subtalar joint motion and alignment during functional activities. In contrast, WBCT offers a dynamic and more accurate representation of how the subtalar joint behaves under load, providing critical insights into the potential for joint subluxation and other deformities that may not be evident in non-weightbearing imaging. Therefore, WBCT can be seen as a more physiologically relevant modality, particularly when assessing conditions such as hindfoot valgus malalignment, where the effects of weightbearing are crucial in understanding the joint’s true alignment and potential for degenerative changes.

Previous studies have simulated weightbearing CT in a supine position using special apparatuses to evaluate symptomatic flatfoot deformity [25,30,31]. These studies utilized partial weight loads ranging from 10% to 50% of body weight. Imaging under partial weightbearing may underestimate subtalar joint subluxation, potentially leaving minor joint changes undetected. Additionally, the supine position may negate the gastrocnemius muscle pull, further reducing subtalar joint subluxation. This study’s results, obtained using upright, full weightbearing imaging under physiological conditions, demonstrate a more precise depiction of subtalar joint changes in patients with hindfoot valgus malalignment.

The hindfoot alignment angle (HAA) assesses the calcaneus’ position relative to the longitudinal tibial axis. Traditionally, this is measured by drawing an angular line connecting two mid-diaphyseal points of the tibia, spaced 50 mm apart and extending the line distally [29,34,35]. While feasible in hindfoot alignment radiographs, CT coronal images often lack the adequate visualization of the tibial diaphysis to define these points. Instead, this study utilized HVA in CT, based on a perpendicular line to the distal tibial joint surface. Although the HVA showed significant increases in WBCT compared to NWBCT for patients with hindfoot valgus, it may not fully replace HAA as a measurement tool. WBCT provides a clinically relevant and reproducible alternative for assessing hindfoot alignment, supported by emerging research [23,36].

There are three established methods for measuring hindfoot alignment: HAA, hindfoot alignment ratio (HAR) and hindfoot moment arm (HMA) [21]. In this study, we measured hindfoot alignment separate from angle and ratio on the hindfoot alignment view. In this study, we measured hindfoot alignment separate from angle and ratio in the hindfoot alignment view. Hindfoot alignment is assessed by both angle and ratio in the hindfoot alignment view, which allows for the evaluation of both angulation and translation due to variations in hindfoot deformities. For instance, hindfeet with minimal valgus angulation but large translation may exhibit significant subtalar joint changes. Thus, subtalar joint changes during upright full weightbearing were evaluated according to two hindfoot alignment view parameters (HAA and HAR). HAA is straightforward to measure and demonstrates high intra- and inter-observer reliability, making it a preferred parameter for routine clinical practice, and while HAR provides additional insights into hindfoot proportional relationships, its complexity limits clinical application. HMA was not included in this study due to the insufficient validation of its utility and reliability [21,37].

This study evaluated the utility of two hindfoot alignment view measurements for identifying talocalcaneal osseous contact in patients with hindfoot valgus malalignment. A prior cadaver study reported a mean cartilage thickness of 0.98 ± 0.16 mm in the talar-subtalar joint [27]. Based on this value, talocalcaneal osseous contact related to sinus tarsi bony impingement was defined as a talocalcaneal distance of less than 2 mm at the Gissane angle in WBCT. The HAA demonstrated the highest accuracy for predicting talocalcaneal osseous contact, with a 73% sensitivity and an 81.8% specificity using a cutoff value of >9.25°. This underscores HAA’s clinical utility in resource-limited settings without WBCT access. However, the ROC analysis also highlights that identifying talocalcaneal osseous contact through radiographic measurements may not detect symptomatic talocalcaneal osseous impingement directly. Nevertheless, the early detection of suspected cases through this method is valuable, as it may prevent the progression of adjacent sclerosis or cystic changes. Although HAR showed weaker predictive power compared to HAA [28], it remains a statistically relevant parameter for assessing hindfoot valgus alignment. Combining these measurements enhances diagnostic accuracy, facilitating the early detection of subtalar joint pathologies and preventing severe deformities.

The study also demonstrated that only the talocalcaneal distance at the Gissane angle in WBCT, indicative of sinus tarsi bony impingement, correlated with hindfoot alignment view parameters. Most previous studies reported that talocalcaneal osseous contact occurs in patients with severe, painful flatfoot deformities [1,3,18,24,25,30]. However, Kim et al. [18] established radiographic cutoff values for predicting lateral bony impingement in progressive collapsing foot deformity (PCFD). Their findings revealed strong correlations between talonavicular coverage angle (TNC) and hindfoot moment arm (HMA) with talocalcaneal and calcaneofibular distances on WBCT, respectively. In contrast, this study’s upright, full weightbearing imaging showed that talocalcaneal osseous contact could occur in hindfoot valgus malalignment patients independently of subtalar subluxation or calcaneofibular impingement.

This study has several limitations. Its retrospective design and single-center setting may limit the generalizability of the findings to broader populations. The small sample size, especially among patients with both NWBCT and WBCT scans, reduces the statistical power of the analyses. In addition, a more thorough analysis of data dispersion, which was not performed in this study, could influence the choice of statistical tests and should be considered in future research. Future studies with larger, multicenter cohorts are needed to validate these correlations and refine radiographic thresholds. Although patient inclusion in this study was based on the presence of hindfoot valgus malalignment (valgus angle > 5°), the cohort was heterogeneous with respect to underlying etiologies, including flatfoot deformity, subtalar joint instability, ligamentous laxity and posterior tibial tendon insufficiency [38]. While the focus of this study was on the common structural manifestation of hindfoot valgus malalignment regardless of cause, we acknowledge that this etiological diversity may influence the interpretation of our findings. Future studies should aim to investigate more etiologically homogeneous patient groups to enhance the accuracy, clinical applicability and generalizability of the results. Regarding the selection of imaging modalities, some patients underwent WBCT alone, while others underwent both WBCT and NWBCT. This decision was based on clinical considerations and the availability of imaging resources. Some patients did not require NWBCT, as their clinical presentation did not warrant additional non-weightbearing imaging, or there were logistical constraints related to imaging schedules. The inclusion of both modalities in certain patients allows for a more comprehensive comparison of weightbearing and non-weightbearing conditions. However, future studies should aim to standardize imaging protocols to avoid inconsistencies and improve comparability.

## 5. Conclusions

This study demonstrates the significant impact of weightbearing on subtalar joint alignment in patients with hindfoot valgus malalignment and highlights the limitations of non-weightbearing imaging modalities. The strong correlations between standard radiographic parameters and WBCT findings underscore the potential of simple radiographs as cost-effective screening tools for evaluating subtalar joint conditions. Identifying clinically relevant cutoff values for the hindfoot alignment angle (HAA) offers a practical framework for diagnosing sinus tarsi bony impingement and other pathologies associated with hindfoot valgus deformity. However, the findings should be interpreted with caution due to the lack of clinical characterization, which limits the generalizability of these results. The recommendations to adopt weightbearing imaging modalities and integrate radiographic thresholds into clinical practice primarily apply to symptomatic patients or those with more severe hindfoot valgus, particularly in cases involving ligamentous laxity or suspected subtalar joint involvement. In these cases, WBCT provides more accurate information than NWBCT, revealing differences in alignment and helping to identify subtalar joint pathologies. Furthermore, the HAA has been shown to be a reliable predictor of subtalar joint involvement in these cases. While WBCT can be a valuable tool, its application should be considered based on the clinical context, and further studies with clinical data are needed to validate these findings and refine the guidelines for its use.

## Figures and Tables

**Table 1:** Inter- and intraobserver reliabilities of radiographic parameters.

Radiographic Parameters	Interobserver Reliability	Intraobserver Reliability
	(ICC, 95% CI)	(ICC, 95% CI)
Hindfoot alignment view		
Hindfoot alignment angle	0.85 (0.73–0.92)	0.87 (0.77–0.93)
Hindfoot alignment ratio	0.93 (0.86–0.97)	0.96 (0.92–0.98)
Computed tomography (CT)		
Heel valgus angle	0.79 (0.58–0.92)	0.82 (0.63–0.91)
Talocalcaneal distance at the Gissane angle	0.88 (0.77–0.94)	0.94 (0.87–0.97)
Subtalar joint subluxation	0.92 (0.83–0.96)	0.95 (0.91–0.97)
Calcaneofibular distance	0.92 (0.83–0.95)	0.94 (0.82–0.97)

ICC, intra-class correlation coefficients. The 95% confidence intervals are shown in parentheses.

**Table 2:** Quantitative analysis of radiographic parameters in non-weightbearing CT (NWBCT) and weightbearing computed tomography (WBCT).

	NWBCT	WBCT	*p*-Value
Heel valgus angle (°)	16.8 ± 4.4	23.2 ± 4.9	<0.001 *
Talocalcaneal distance at the angle Gissane (mm)	4.6 ± 1.3	1.7 ± 1.2	<0.001 *
Subtalar joint subluxation (mm)	3.7 ± 2.6	5.5 ± 2.7	<0.001 *
Calcaneofibular distance (mm)	6.5 ± 2.5	5.2 ± 2.2	0.002 *

Data are mean values with standard deviations. * *p* < 0.05 was considered statistically significant.

**Table 3:** Pearson’s correlation coefficients of radiographic parameters in the hindfoot alignment view and WBCT.

Hindfoot Alignment View	Radiographic Measurements on WBCT			
	HVA	TCD	SL	CF
Hindfoot alignment angle	0.53 (0.002) *	0.48 (<0.001) *	0.18 (0.148)	0.22 (0.074)
Hindfoot alignment ratio	0.71 (<0.001) *	0.25 (0.03) *	0.17 (0.358)	0.06 (0.641)

Pearson’s correlation coefficients (*p*-value); HVA, heel valgus angle; TCD, talocalcaneal distance at the Gissane angle; SL, subtalar joint subluxation; CF, calcaneofibular distance; * *p* < 0.05 was considered statistically significant.

## Data Availability

The datasets used and/or analyzed during the current study are available from the corresponding author upon reasonable request.
